# Stall in Heart Disease Death Rates, Evidence From Maine, 1999–2017

**DOI:** 10.5888/pcd17.190405

**Published:** 2020-08-20

**Authors:** Jennifer A. Sinatra, Sara L. Huston

**Affiliations:** 1 Epidemic Intelligence Service, Division of Scientific Education and Professional Development, Centers for Disease Control and Prevention, Atlanta, Georgia; 2 Maine Department of Health and Human Services, Augusta, Maine; 3 University of Southern Maine, Portland, Maine

## Abstract

**Introduction:**

Since the 1950s, heart disease deaths have declined in the United States, but recent reports indicate a plateau in this decline. Heart disease death rates increased in Maine from 2011–2015. We examined reasons for the trend change in Maine’s heart disease death rates, including the contributing types of heart disease.

**Methods:**

We obtained Maine’s annual heart disease death data for 1999–2017 from CDC’s Wide-ranging Online Data for Epidemiologic Research (CDC WONDER). We used joinpoint regression to determine changes in trend and annual percentage change (APC) in death rates for heart disease overall and by demographic groups, types of heart disease, and geographic area.

**Results:**

Joinpoint modeling showed that Maine’s age-adjusted heart disease death rates decreased during 1999–2010 (−4.2% APC), then plateaued during 2010–2017 (−0.1% APC). Death rates flattened for both sexes and age groups ≥45 years. Although death rates for acute myocardial infarction (AMI) decreased through 2017, hypertensive heart disease (HHD) and heart failure death rates increased. Death rates attributable to diabetes-related heart disease and non-AMI ischemic heart disease (IHD) plateaued.

**Conclusion:**

Declines in Maine’s heart disease death rates have plateaued, similar to national trends. Flattening rates appear to be driven by adverse trends in HHD, heart failure, diabetes-related heart disease, and non-AMI IHD. Increased efforts to address cardiovascular disease risk factors, chronic heart disease, and access to care are necessary to continue the decrease in heart disease deaths in Maine.

**Summary**

**What is already known on this topic?**

Heart disease death rates in the United States have declined substantially since the 1950s, but they have declined more slowly or stalled in recent years.

**What is added by this report?**

In Maine, heart disease death rates stalled from 2011 through 2017. Rates plateaued in all age groups >45 years. Adverse trends in hypertensive heart disease, heart failure disease, diabetes-related heart disease, and ischemic heart disease (not including acute myocardial infarction) appeared to drive the plateauing rates.

**What are the implications for public health practice?**

These findings indicate that increased public health messaging and interventions emphasizing prevention and control of hypertension, obesity, tobacco use, and diabetes are necessary to reverse this change in heart disease death rates.

## Introduction

The decline in heart disease death rates in the United States during the latter part of the 20th century is considered a major public health achievement (1). Nationally, age-adjusted death rates for heart disease decreased by 56% during 1950–1996 (1), and by 30% during 2000–2010 (2). However, heart disease continues to be the leading cause of death in the United States (3). Since 2010, the decline in heart disease death rates has slowed nationally; during 2010–2013, the annual decline in heart disease slowed to 1.4% from a 3.9% decline during 2000–2010 (2,4–6).

There are many possible reasons for the major declines in death rates attributable to heart disease. Large decreases in smoking, a decrease in mean blood pressure levels, an increase in hypertension treatment, and the use of evidence-based medical treatment all played a role. Public health agencies and initiatives educated medical professionals and the public on blood pressure and cholesterol management, smoking cessation, treatment strategies, and how to involve the community in decreasing risk factors (1,2,7). It is important to monitor heart disease death rates both nationwide and at the state and county levels to ensure these successes continue.

Maine’s age-adjusted heart disease death rate has typically been lower than that of the United States, and Maine was one of the first states where heart disease deaths declined below cancer deaths (8). In Maine, routine surveillance data review (unpublished) identified that heart disease death rates increased significantly from 2011 to 2015. We examined Maine’s heart disease death rates during 1999–2017 to further characterize trends in recent heart disease death rates, whether the change in death rates was experienced by all demographic groups equally, which types of heart disease were contributing to the flattening, whether the flattening occurred in all geographic regions of Maine, and whether regions with higher death rates had a different rate of decline that those with lower death rates. 

## Methods

### Data source

We investigated heart disease death rates among all people in Maine during 1999–2017. We analyzed trends in heart disease death rates by sex, age, type of heart disease, Maine public health district of residence, and urbanicity. Data were retrieved from Centers for Disease Control and Prevention’s Wide-ranging Online Data for Epidemiologic Research (CDC WONDER; https://wonder.cdc.gov/) mortality database, which contains annual mortality data collected from death certificates and compiled by the National Center for Health Statistics at CDC (9). We used underlying cause of death and multiple cause of death databases in the analysis. These databases contain demographic data that include residence, race, ethnicity, and sex. The underlying cause of death database includes data from the single underlying cause of death identified from the death certificate; the multiple cause of death database includes data from the single underlying cause of death and additional multiple causes. More than 99% of deaths occurring in the United States are believed to be registered (9).

Underlying and multiple causes of death were defined by the *International Classification of Diseases*, *10th Revision* (Table 1). Heart diseases analyzed from the underlying cause of death database included all heart disease; hypertensive heart disease (HHD); acute myocardial infarction (AMI); ischemic heart disease (IHD) not including AMI; pulmonary heart disease; cardiac arrest; heart failure; and complications and ill-defined heart disease. Diseases from the multiple cause of death database include heart failure–related heart disease, diabetes-related heart disease, and 2 additional cardiac arrest definitions. To try to eliminate cardiac arrest deaths that may not truly have been related to heart disease, we also used the Million Hearts cardiac arrest definition (10) and examined cardiac arrest deaths that had heart disease identified as a multiple cause of death.

**Table 1:** *International Classification of Diseases, Tenth Revision* (ICD-10) Codes Used in Analysis, Trends in Heart Disease Death Rates in Maine, 1999–2017

Category	ICD-10 Codes
**Underlying cause of death**	
All heart disease	I00–I02, I05–I09, I11, I13, I20–I51
Hypertensive heart disease	I11, I13
Acute myocardial infarction	I21
Ischemic heart diseases not including acute myocardial infarction	I20, I22–I25
Pulmonary heart disease	I26–I28
Cardiac arrest	I46
Heart failure	I50
Complications and ill-defined heart disease	I51
**Multiple causes of death**	
Heart failure–related heart disease	Underlying: I00–I02, I05–I09, I11, I13, I20–I51
	Contributing: I50
Diabetes-related heart disease	Underlying: I00–I02, I05–I09, I11, I13, I20–I51
	Contributing: E10–E14
Million Hearts cardiac arrest definition	Underlying: I46
	Contributing: I10–I11, I12.9, I13.0, I13.9, I20–I21, I24, I50, I60–I69, I70, I71.3, I71.4, I73.9, G45.0, G45.1, G45.2, G45.8, G45.9
Cardiac arrest with heart disease contributing	Underlying: I46
	Contributing: I00–I02, I05–I09, I11, I13, I20–I45, I47–I51

### Statistical analysis

We used the underlying cause of death database to calculate age-adjusted heart disease death rates per 100,000 people and by sex and public health district of residence. We used both the underlying cause of death database and the multiple cause of death database to calculate age-adjusted death rates by type of heart disease (Table 2). Age-adjusted rates were standardized using the 2000 US Census Bureau’s standard population. We also calculated age-specific (crude) heart disease death rates for age groups 25–44, 45–64, 65–84, and ≥85 years. Standard errors were calculated for all rates. Maine has 16 counties and 8 public health districts (made up of 1 or more counties), which are the primary division of Maine’s public health infrastructure and the level at which many programs are implemented (11). Public health districts were used as a geographic division instead of counties both for this reason and because their larger populations produce more statistically reliable estimates. In CDC WONDER, the 2013 National Center for Health Statistics Urban-Rural Classification Scheme for Counties classifies counties by urbanicity (12). We used these classifications to examine age-adjusted death rates in metropolitan and nonmetropolitan populations in Maine to see findings within larger populations than the health districts. Data for nonwhite race categories and Hispanic or Latino ethnicities were statistically unreliable because of the limited numbers of deaths among these groups (Maine’s population is approximately 95% white) (13).

**Table 2:** Heart Disease Death Rates Using Underlying Cause of Death Database, by Selected Demographics and Disease Types, Maine, 2017

Characteristic	No. of Deaths	Age-Adjusted Death Rate Per 100,000 People (Standard Error)
**Total**	2,844	143.5 (2.7)
**Sex**		
Female	1,311	111.6 (3.2)
Male	1,533	183.3 (4.8)
**Age, y^a^**		
<25	12	—b
25–44	171	13.9 (1.1)
45–64	1,702	105.7 (2.6)
65–84	4,739	533.0 (7.7)
≥85	4,911	3,814.0 (54.4)
**Disease**		
Cardiac arrest^c^	19	—b
AMI	449	22.7 (1.1)
Hypertensive heart disease	143	7.4 (0.6)
Heart failure–related^d^	1,160	58.7 (1.7)
Diabetes-related^d^	259	13.2 (0.8)
Ischemic heart diseases not including AMI	1,051	52.2 (1.6)
Pulmonary heart disease	71	3.7 (0.4)
Complications and ill-defined heart disease	177	8.8 (0.7)

Abbreviations: —, unreliable; AMI, acute myocardial infarction; ICD-10, International Classification of Diseases, 10th Revision.

a 2014–2017, crude death rate per 100,000 people. Used multiple years because of fewer than 10 deaths per year among those aged <25 y, which would have required the data be suppressed for privacy reasons.

b Unreliable because rate calculated with a numerator of 20 or less.

c Defined using ICD-10 code number I46.

d Used multiple cause of death database.

We used Joinpoint 4.6.0.0 analytic software, available from the National Cancer Institute, to perform trend analyses on heart disease death rates (14). Joinpoint assesses trends over time using joinpoint regression, which fits a series of joined straight lines to the data. The lines start and end when a significant change in trend is detected, with that change represented by a joinpoint. The software runs multiple models based on the number of years of data. The null model shows no change in trend, the second model finds one joinpoint, the third finds two, and so on. The default maximum number of joinpoints is based on having at least seven data points to consider allowing a joinpoint, and on average, at least two data points between consecutive joinpoints. Because we analyzed 18 years of data, the default maximum number of joinpoints tested was 3. The best overall model with the smallest number of joinpoints was found using the permutation test. The program calculates the annual percentage change (APC) for each segment identified and determines whether it is significantly different from zero (2-tailed *P* ≤ .05). We classified changes in trend as decreasing (ie, where the most recent APC is significantly decreasing), increasing (ie, where the most recent APC is significantly increasing), or flattening (ie, where the most recent APC is nonsignificant in either the increasing or decreasing direction). This project was reviewed for human subjects protection by CDC and determined to be nonresearch.

## Results

Numbers of heart disease deaths and death rates in 2017 are presented by selected demographics and disease types in Table 2 for context; however, outcomes in this study are the APC in heart disease death rate.

### Heart disease mortality trends in Maine

**Figure 1:**
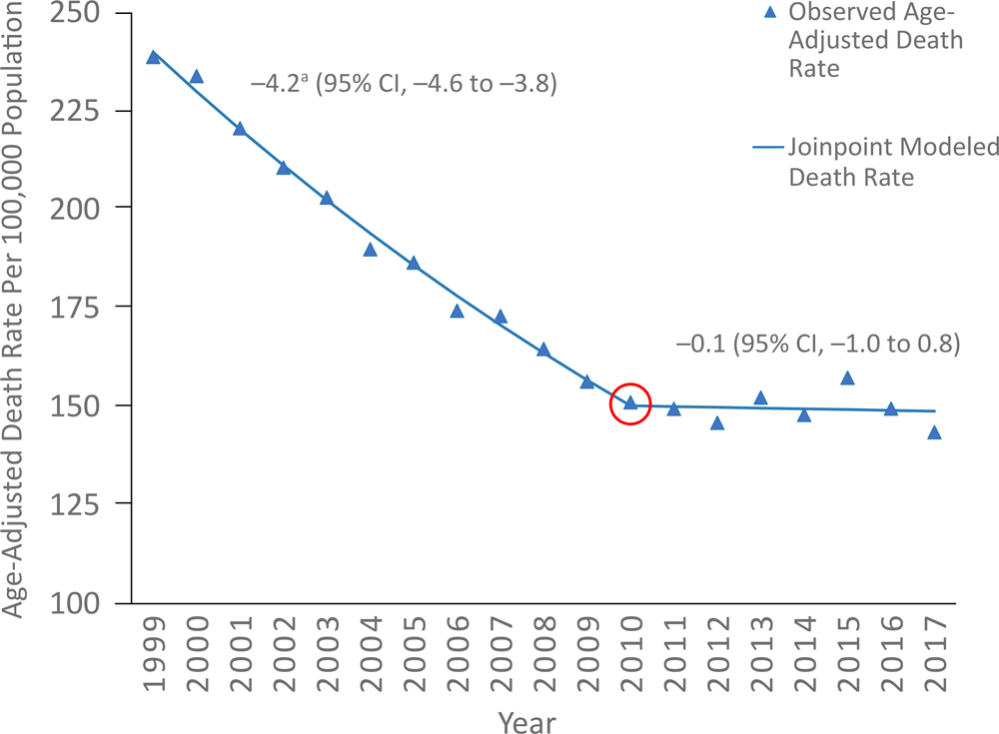
Comparison of observed and joinpoint-modeled age-adjusted heart disease death rates, Maine, 1999–2017. Death rates are age-adjusted to the 2000 US standard population. *International Classification of Diseases, 10th Revision* (ICD-10) codes used: I00-I02, I05–I09, I11, I13, I20–I25, I26–I28, I30–I51. ^a^ Annual percentage change from 1999–2010 is significantly different from 0 at an alpha level of .05. Data source: CDC WONDER.

In Maine, the APC for age-adjusted heart disease death rates changed by −4.2% (95% confidence interval [CI], –4.6% to −3.8%) during 1999–2010, but then plateaued to −0.1% (95% CI, −1.0% to 0.8%) during 2010–2017 (Figure 1).

**Table:** 

Year	Observed Age-Adjusted Death Rate Per 100,000 Population	Joinpoint Modeled Death Rate Per 100,000 Population
1999	238.72	239.95
2000	233.86	229.95
2001	220.65	220.36
2002	210.61	211.18
2003	203.03	202.38
2004	189.89	193.94
2005	186.53	185.86
2006	174.31	178.12
2007	172.97	170.69
2008	164.63	163.58
2009	156.37	156.76
2010	151.1	150.23
2011	149.46	150.03
2012	145.9	149.83
2013	152.27	149.63
2014	147.89	149.43
2015	157.31	149.24
2016	149.51	149.04
2017	143.48	148.84

### Heart disease mortality trends in Maine, by demographic group

**Figure 2:**
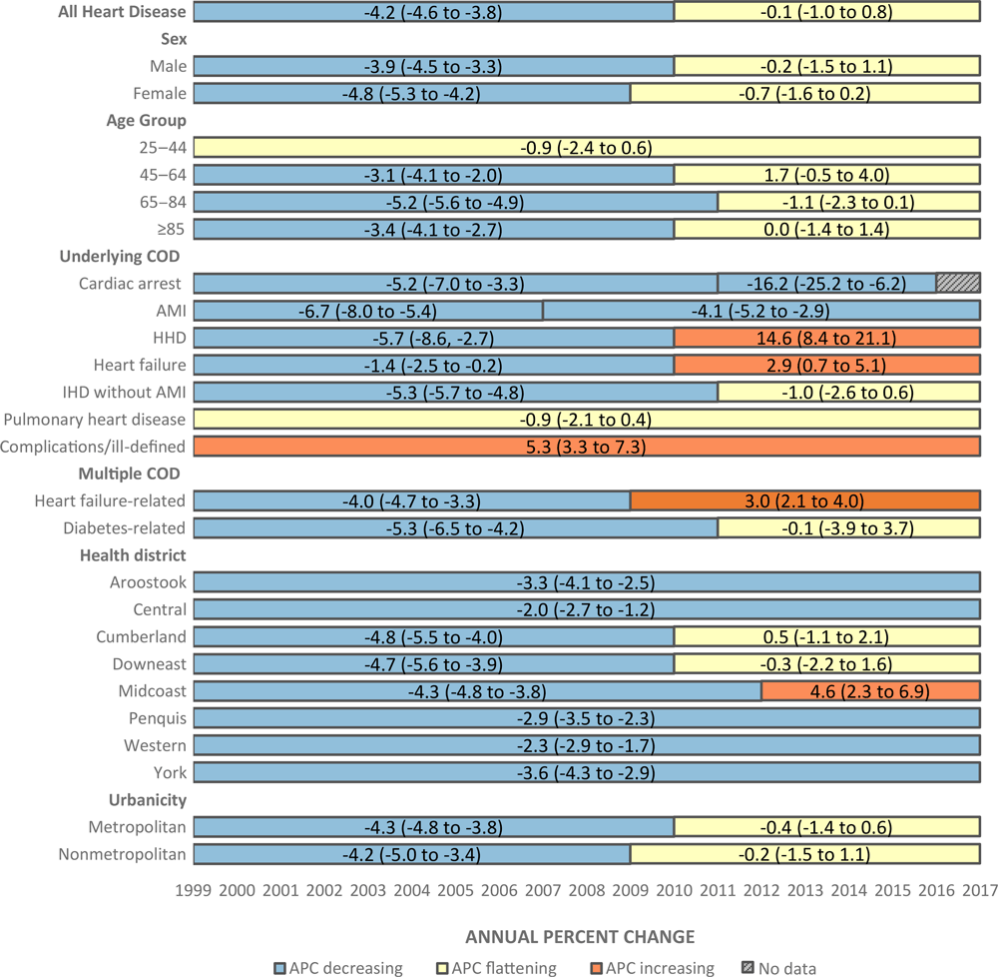
Heart disease mortality trends in Maine, by sex, age group, and type of heart disease, 1999–2017. All trends except age group determined using age-adjusted death rates. Data in bars are APC (95% CI). 95% CIs are provided to give the reader an indication of statistical stability and should not be used to determine whether changes in trend occurred. Significant changes in trend (increasing or decreasing) were determined by using joinpoint modeling to identify where APCs were significantly different from zero at the alpha = 0.05 level. There were fewer than 20 cases of cardiac arrest in 2017, so the death rate for that year was unreliable. Abbreviations: AMI, acute myocardial infarction; APC, annual percentage change; CI, confidence interval; COD, cause of death; HHD, hypertensive heart disease; ICD-10, International Classification of Diseases, 10th revision; IHD, ischemic heart disease; NA, not available.

Although men had higher age-adjusted heart disease death rates than women during 1999–2017, death rates plateaued for both sexes during the latter part of this period. APC in death rate for men changed by −3.9% (95% CI, −4.5% to −3.3%) during 1999–2010 and then flattened at −0.2% (95% CI, −1.5% to 1.1%) during 2010–2017. APC for women changed by −4.8% (95% CI, −5.3% to −4.2%) during 1999–2009 and then plateaued at −0.7% (95% CI, −1.6% to 0.2%) during 2009–2017 (Figure 2).

**Table:** 

Category	Period 1 Dates	Period 1 APC (95% CI)	Period 2 Dates	Period 2 APC (95% CI)
Heart Disease	1999–2010	–4.2 (–4.6 to –3.8)^a^	2010–2017	–0.1 (–1.0 to 0.8)
**Sex**				
Male	1999–2010	–3.9 (–4.5 to –3.3)^a^	2010–2017	–0.2 (–1.5 to 1.1)
Female	1999–2009	–4.8 (–5.3 to –4.2)^a^	2009–2017	–0.7 (–1.6 to 0.2)
**Age, y**				
25–44	1999–2017	–0.9 (–2.4 to 0.6)	NA	NA
45–64	1999–2010	–3.1 (–4.1 to –2.0)^a^	2010–2017	1.7 (–0.5 to 4.0)
65–84	1999–2011	–5.2 (–5.6 to –4.9)^a^	2011–2017	–1.1 (–2.3 to 0.1)
≥85	1999–2010	–3.4 (–4.1 to –2.7)^a^	2010–2017	0.0 (–1.4 to 1.4)
**Underlying cause of death**				
Cardiac arrest	1999–2011	–5.2 (–7.0 to –3.3)^a^	2011–2016	–16.2 (–25.2 to –6.2)^a^
AMI	1999–2007	–6.7 (–8.0 to –5.4)^a ^	2007–2017	–4.1 (–5.2 to –2.9)^a^
HHD	1999–2010	–5.7 (–8.6 to –2.7)^a^	2010–2017	14.6 (8.4 to 21.1)^a^
Heart failure	1999–2010	–1.4 (–2.5 to –0.2)^a^	2010–2017	2.9 (0.7 to 5.1)^a^
IHD without AMI	1999–2011	–5.3 (–5.7 to –4.8)^a^	2011–2017	–1.0 (–2.6 to 0.6)
Pulmonary heart disease	1999–2017	–0.9 (–2.1 to 0.4)	NA	NA
Ill-defined heart disease	1999–2017	5.3 (3.3 to 7.3)^a^	NA	NA
**Multiple cause of death**				
Heart failure–related	1999–2009	–4.0 (–4.7 to –3.3)^a^	2009–2017	3.0 (2.1 to 4.0)^a^
Diabetes-related	1999–2011	–5.3 (–6.5 to –4.2)^a^	2011–2017	–0.1 (–3.9 to 3.7)
**Health district**				
Aroostook	1999–2017	–3.3 (–4.1 to –2.5)^a^	NA	NA
Central	1999–2017	–2.0 (–2.7 to –1.2)^a^	NA	NA
Cumberland	1999–2010	–4.8 (–5.5 to –4.0)^a^	2010–2017	0.5 (–1.1 to 2.1)
Downeast	1999–2010	–4.7 (–5.6 to –3.9)^a^	2010–2017	–0.3 (–2.2 to 1.6)
Midcoast	1999–2012	–4.3 (–4.8 to –3.8)^a^	2012–2017	4.6 (2.3 to 6.9)^a^
Penquis	1999–2017	–2.9 (–3.5 to –2.3)^a^	NA	NA
Western	1999–2017	–2.3 (–2.9 to –1.7)^a^	NA	NA
York	1999–2017	–3.6 (–4.3 to –2.9)^a^	NA	NA
**Rural/urban status**				
Metropolitan	1999–2010	–4.3 (–4.8 to –3.8)^a^	2010–2017	–0.4 (–1.4 to 0.6)
Nonmetropolitan	1999–2009	–4.2 (–5.0 to –3.4)^a^	2009–2017	–0.2 (–1.5 to 1.1)

^a^ APC is significantly different from zero at an alpha level of .05.

Since 2010, all age groups ≥45 years had significant changes in age-specific heart disease death rate trends, with the decrease flattening during this period. The rate for those aged 25–44 years did not have a significant change in trend during 1999–2017, and APC was steady at −0.9% (95% CI, −2.4% to 0.6%). APC for the group aged 45–64 years changed by −3.1% (95% CI, −4.1% to −2.0%) during 1999–2010, and then flattened at 1.7% (95% CI, −0.5% to 4.0%) during 2010–2017. APC for the group aged 65–84 years changed by −5.2% (95% CI, –5.6% to −4.9%) during 1999–2011 and then flattened at −1.1% (95% CI, −2.3% to 0.1%) during 2011–2017. APC for the group aged ≥85 years changed by −3.4% (95% CI, −4.1% to −2.7%) during 1999–2010, and then flattened at 0.0% (95% CI, −1.4% to 1.4%) during 2010–2017 (Figure 2).

### Heart disease mortality trends in Maine, by type of disease

When defining cardiac arrest using the I46 code, age-adjusted death rates changed by −5.2% (95% CI, −7.0% to −3.3%) during 1999–2011 and then accelerated to a −16.2% change (95% CI, −25.2% to −6.2%) during 2011–2016, but they were unreliable for 2017 because of the limited number of deaths reported. Limiting cardiac arrest deaths to those defined as such by Million Hearts or to those defined as heart disease by using the multiple cause of death database (Table 1) excluded most of the Maine heart disease deaths to the point that all yearly data were suppressed because of low numbers of deaths reported. AMI age-adjusted death rates continued to decrease during 1999–2017 but had significant changes in the rate of decrease. AMI changed by −6.7% (95% CI, −8.0% to −5.4%) during 1999–2007 but slowed to a −4.1% change (95% CI, −5.2% to −2.9%) during 2007–2017 (Figure 2).

Age-adjusted death rate trends for HHD, heart failure, and heart failure–related heart disease changed from decreasing to increasing during 1999–2017. HHD changed by −5.7% (95% CI, −8.6% to −2.7%) during 1999–2010, and increased 14.6% (95% CI, 8.4% to 21.1%) during 2010–2017. Heart failure changed by −1.4% (95% CI, −2.5% to −0.2%) during 1999–2010, and increased 2.9% (95% CI, 0.7% to 5.1%) during 2010–2017. Heart failure–related heart disease changed by −4.0% (95% CI, −4.7% to −3.3%) during 1999–2009, and increased by 3.0% (95% CI, 2.1% to 4.0%) during 2009–2017 (Figure 2).

Age-adjusted death rates for both diabetes-related heart disease and IHD not including AMI significantly changed from decreasing to plateauing during 1999–2017. Diabetes-related heart disease changed by −5.3% (95% CI, −6.5% to −4.2%) during 1999–2011, and then flattened at −0.1% (95% CI, −3.9% to 3.7%) during 2011–2017. IHD not including AMI changed by −5.3% (95% CI, −5.7% to −4.8%) during 1999–2011, and then flattened at −1.0% (95% CI, −2.6% to 0.6%) during 2011–2017 (Figure 2).

The age-adjusted death rate for pulmonary heart disease was stable during 1999–2017 (APC, −0.9% [95% CI, −2.1% to 0.4%]). APC for complications and ill-defined heart disease did not change significantly (APC, 5.3% [95% CI, 3.3% to 7.3%]). Chronic rheumatic heart disease and endocarditis death rates were suppressed or unreliable for some years because of limited deaths reported, and a joinpoint analysis could not be done.

### Geographic differences in heart disease in Maine

Joinpoints indicating a significant change in age-adjusted heart disease death rate trend were found in 3 of Maine’s 8 public health districts (Downeast, Midcoast, and Cumberland) during 1999–2017. Of these, only the Midcoast district changed from a decreasing death rate during 1999–2012 (APC, −4.3% [95% CI, −4.8% to −3.8%]) to an increasing death rate during 2012–2017 (APC, 4.6% [95% CI, 2.3% to 6.9%]). The Cumberland and Downeast health districts changed from decreasing death rates to plateauing death rates (Figure 2). The remaining 5 public health districts (Aroostook, Central, Penquis, Western, and York) all had significantly declining APCs with no joinpoint during 1999–2017 (Figure 2). Age-adjusted heart disease death rates in 2017 ranged from 125.8 deaths per 100,000 people in York District to 164.2 deaths per 100,000 people in Penquis District (Table 3). The highest death rates did not correspond with the largest changes in APC.

**Table 3:** Maine Health District Geography, Population, and Population Density, Trends in Heart Disease Death Rates in Maine, 1999–2017

Health District	Counties	Region	Population, 2017	Largest City	Population Density Per Square Mile, 2017	No. of Deaths Due to Heart Disease, 2017	2017 Age-Adjusted Heart Disease Death Rate Per 100,000 People (SE)
Aroostook	Aroostook	Northern inland	67,595	Presque Isle	10.1	181	153.3 (11.8)
Central	Kennebec, Somerset	Central inland	172,350	Augusta	36.0	368	147.4 (7.8)
Cumberland	Cumberland	Southwest coastal	292,344	Portland	350.0	508	126.8 (5.7)
Downeast	Hancock, Washington	Eastern coastal	86,137	Ellsworth	20.8	223	154.8 (10.7)
Midcoast	Knox, Lincoln, Sagadahoc, Waldo	Midcoast	149,282	Bath	82.7	404	162.0 (8.3)
Penquis	Penobscot, Piscataquis	Central inland	167,991	Bangor	22.8	389	164.2 (8.5)
Western	Androscoggin, Franklin, Oxford	Western inland	194,851	Lewiston	45.9	390	141.1 (7.3)
York	York	Southwest coastal	204,513	Biddeford	206.4	381	125.8 (6.6)

Abbreviation: SE, standard error.

Death rates decreased significantly from 1999–2010 in metropolitan areas (APC, −4.3% [95% CI, −4.8 to −3.8]) and then flattened during 2010–2017 (APC, −0.4% [95% CI, −1.4 to 0.6]). Death rates also decreased significantly in nonmetropolitan areas from 1999–2009 (APC, −4.2% (95% CI, −5.0 to −3.4)] and then flattened during 2009–2017 (APC, −0.2% [95% CI, −1.5 to 1.1]).

## Discussion

We found that after decades of decline, overall heart disease death rates are no longer declining in Maine. This trend is consistent with other studies that have reported a flattening in heart disease mortality nationally after decades of improvement (2–4). Death rates in Maine flattened for both sexes and in all age groups ≥45 years. This adverse change in trend appeared to be driven by increases in rates of death attributable to HHD and heart failure as well as a flattening of the decline in diabetes-related heart disease and IHD other than AMI. We found that AMI death rates continued to decrease, although at a slower rate.

Age-adjusted heart disease death rates were no longer declining in 3 of 8 public health districts. Rates increased in the Midcoast district and plateaued in the Cumberland and Downeast districts. The Midcoast district is coastal and moderately populated, the Downeast district is a coastal rural area, and the Cumberland district includes Portland, Maine’s largest city (Table 3). Rates continued to decline in the Aroostook, Central, Penquis, Western, and York districts. The Aroostook health district is in the northernmost part of Maine and is rural; just south is the Penquis district, which is also mostly rural, but includes the city of Bangor. The Central and Western districts are generally rural but include the cities of Augusta and Lewiston, respectively. The York health district is along the coast and is the second most-densely populated area of Maine (15). Both the Aroostook and Penquis districts had comparatively higher rates of heart disease death that were still declining, whereas the flattening and increasing trends included coastal health districts (Downeast and Midcoast), which also had relatively high age-adjusted rates of heart disease deaths.

Because populations in some health districts were small, we also compared metropolitan and nonmetropolitan populations overall, which provided larger population numbers. Heart disease death rates changed from decreasing to flattening in 2010 for the metropolitan population and in 2009 for the nonmetropolitan population. This finding was different from what was reported in some rural health districts, which showed that heart disease death rates were still declining. Calculating heart disease death rates for the entire nonmetropolitan population of Maine might be a more accurate representation of what was occurring, compared with examining smaller rural areas.

We cannot show a single definitive cause for the flattening of heart disease mortality in Maine, but the types of heart diseases we found to be driving the change may provide some clues for overall national trends and further research in Maine and the United States. Studies that are able to examine trends in disease incidence and case fatality for each type of heart disease could better determine why heart disease mortality has flattened in Maine and the United States. Increases in cardiovascular disease risk factors, including obesity, hypertension, and diabetes, have been widely recognized since at least the 1990s (16–18). Obesity prevalence among Maine adults increased from 19.4% in 1999 to 29.1% in 2017 (19). Hypertension prevalence increased from 26.6% in 1999 to 34.8% in 2017 (19). The percentage of Maine adults who received a diagnosis of diabetes was 5.4% in 1999 but approximately doubled to 10.7% in 2016 (19). These risk factors have been targeted by prevention programs for several decades. The slowing of the decline in heart disease death rates may indicate either the limitations of prevention programs or that certain populations are not being reached by public health interventions.

Decreases in heart disease death rates in the latter part of the 20th century occurred because of a combination of primary prevention, including decreased smoking prevalence and reduction of cholesterol and blood pressure levels, and secondary and tertiary prevention, such as improved use of resuscitation, revascularization, and medications (20). The Million Hearts program was established in 2012 as a 5-year national initiative to prevent a million cardiovascular events through promotion of public health efforts and improvements in care. The flattening heart disease death rate and increase in risk factors for cardiovascular morbidity and mortality emphasize the need to continue and expand programs that improve heart disease prevention and treatment (19).

In our analysis, we noted a continued decline in death rate for AMI (although the decline is slowing), but the rate of death attributable to heart failure is increasing. These findings may indicate that patients are now more likely to survive an acute event, but after treatment an increasing number of patients eventually die of heart failure. The increase in death rates attributable to heart failure may also be a result of the increased prevalence of hypertension in Maine (19). Our communities and medical systems are more successfully responding to and treating acute events but may need to improve rehabilitation and follow-up care and ensure access to this care for better long-term outcomes. Thirty-day readmission rates after hospitalization with heart failure were lower with an early follow-up appointment, defined as within 7 days after discharge (21,22). The Million Hearts program reported that people who attend 36 sessions of cardiac rehabilitation have a 47% lower risk for death and a 31% lower risk for heart attack than those who attended 1 session, which illustrates the value of follow-up care. However, only 10% of eligible patients with heart failure were referred for cardiac rehabilitation (23,24).

We also found that deaths reported as cardiac arrest continued to decline. Although reported deaths due to cardiac arrest may be overestimated because some unattended deaths are coded as cardiac arrest, research has shown that more than half of people with cardiac arrest listed as the underlying cause of death have significant cardiac disease (25,26). When we used the multiple cause of death database to define cardiac arrest more specifically by using the Million Hearts definition or by having heart disease as a contributing cause, the majority of Maine’s reported cardiac arrest deaths were excluded. This indicated the number of true cardiac arrest deaths in Maine is low, but we cannot specifically define it or look for trends. Our data do indicate, however, that trends in cardiac arrest are not contributing to the stall in the heart disease death rate decline in Maine.

Vaughan et al found that recent increases in heart disease death rates were reported outside large metropolitan areas, including some of Maine’s rural counties (6). According to 2010 US Census Bureau data, Maine was the most rural state in the United States, with 61% of the population living in rural areas (27). Much of rural Maine is designated as medically underserved by the Health Resources and Services Administration (28). Decreased availability of care may lead to cardiovascular risk factors being undiagnosed or poorly managed and inconsistent follow-up care after an acute heart disease event. Several rural hospitals in Maine have closed since 2010, and others are facing financial challenges or health professional shortages (28). It should be noted that we also observed flattening of the death rate in some of Maine’s more urban areas.

This study has at least 2 limitations. First, data were obtained from death certificates, which may be inaccurate or incomplete when assigning the underlying cause of death. Studies validating the accuracy of death certificates show that deaths from heart disease tend to be overreported and deaths from diabetes tend to be underreported (29,30). We also did not have reliable occupation or education data, because this information is not reliable on death certificates. Having this information may have lent more meaning to the analysis of metropolitan vs nonmetropolitan areas, as education or type of occupation could be important confounders. A future study could use other methods to look at these characteristics in relation to heart disease deaths. The second limitation was Maine’s small population, which leads to a lack of statistical power and even unreliable or suppressed data, especially when looking at smaller geographic areas, smaller populations, or rare types of heart disease.

Through 2017, heart disease death rates were no longer declining in Maine. Continued surveillance will determine if these rates worsen or start to decrease again. Public health messaging to the clinical community and the public, and public health interventions should continue to emphasize the prevention and control of hypertension, obesity, tobacco use, and diabetes, because without improvement in these areas, increases in incidence and prevalence of heart disease in Maine may outweigh gains made in acute care. Although it remains important to improve treatment of acute cardiac events, longer-term management after acute events is crucial to manage heart failure and prevent subsequent heart attacks. Long-term management should include early identification of worsening disease and improving aspects of patient management using community support, education, and rehabilitation.
